# Sense of Coherence as a Mediator in the Association Between Empathy and Moods in Healthcare Professionals: The Moderating Effect of Age

**DOI:** 10.3389/fpsyg.2022.847381

**Published:** 2022-04-25

**Authors:** Miyo Hori, Eisho Yoshikawa, Daichi Hayama, Shigeko Sakamoto, Tsuneo Okada, Yoshinori Sakai, Hideomi Fujiwara, Kazue Takayanagi, Kazuo Murakami, Junji Ohnishi

**Affiliations:** ^1^Foundation for Advancement of International Science, Tsukuba, Japan; ^2^Department of Medical Psychology, Nippon Medical School, Tokyo, Japan; ^3^Department of Neuropsychology, Nippon Medical School, Tokyo, Japan; ^4^Faculty of Commerce, Chuo Gakuin University, Chiba, Japan; ^5^Department of Rehabilitaion, Tsuchiura Kyodo General Hospital, Tsuchiura, Japan; ^6^Department of Gastroenterology, Tsuchiura Kyodo General Hospital, Tsuchiura, Japan; ^7^Tsuchiura Kyodo General Hospital, Tsuchiura, Japan; ^8^Japan Society of Healing Environment, Tokyo, Japan; ^9^Department of Food and Nutrition, Tokyo Kasei University, Tokyo, Japan

**Keywords:** sense of coherence (SOC), empathy, moods, moderated mediation analysis, healthcare professional, age

## Abstract

While empathy is considered a critical determinant of the quality of medical care, growing evidence suggests it may be associated with both one’s own positive and negative moods among healthcare professionals. Meanwhile, sense of coherence (SOC) plays an essential role in the improvement of both psychological and physical health. Reportedly, individual SOC reaches full stability after around age 30. The aim of this study was first to evaluate the mediatory role of SOC on the association between empathy and individual moods among 114 healthcare professionals in a general hospital, and then to examine the moderating effect of age on this association. Participants completed a range of self-report demographic questionnaires, Empathy Process Scale (EPS), the 13-item Antonovsky’s SOC, and Profile of Mood States (POMS). Findings showed that SOC mediated the relations between empathy (EPS) and both POMS-Vigor (POMS-V: self-vigor mood) and POMS-Depression (POMS-D: self-depression mood). Notably, moderated mediation analysis revealed that there was a significant interaction (age × SOC) on self-vigor mood (POMS-V) in healthcare professionals. The indirect effect of empathy (EPS) on self-vigor mood (POMS-V) through SOC was significant at over mean age “32.83.” Although there was no significant interaction with age regarding the indirect effect of empathy (EPS) on self-depression mood (POMS-D), in the sub-category level analysis of empathy (EPS), we found a significant interaction item [age × empathy for other’s negative affect (EPS-N)] on SOC. This indirect effect was also significant at over mean age “32.83.” Taken, together, the current study highlighted the significant mediator of SOC on that empathy amplifies self-vigor mood and attenuates self-depression mood as a protective factor among the Japanese healthcare professionals. Some components of these processes may depend on the moderating role of age, indicating that we may need to consider the SOC development with age for more effective empathy performance interventions among healthcare professionals.

## Introduction

The role of empathy in healthcare professionals, as it relates to therapeutic relationships and quality of care in medical hospitals, has been widely discussed (Chaitoff et al., 2017; Wilkinson et al., 2017; Wang et al., 2018; Menezes et al., 2021; Abdulkader et al., 2022). Evidence suggests that clinicians’ empathy is associated with strong communication with their patients, higher patient satisfaction, better psychosocial adjustment, lower levels of psychological distress, and enhanced ability to obtain patient information (Lelorain et al., 2012; Boissy et al., 2016). Although definitions of empathy are inconsistent, empathy is generally conceptualized as a multi-faceted construct composed of cognitive and affective components. Cognitive empathy involves taking the perspective of others by attempting to understand and mentally visualize another’s point of view and affective states (Davis, 1983; de Waal, 2008; Tibi-Elhanany and Shamay-Tsoory, 2011). Another aspect of cognitive empathy is called empathic accuracy and is the ability to intuit the feelings of others based on their actions and affects (Ickes et al., 1990). Affective empathy is feeling and sharing the affect that another person experiences (Davis et al., 1994), and is also known as empathic concern.

Several lines of evidence have demonstrated that depression is correlated with low cognitive accuracy and empathy, the latter of which is indicated by poor perspective taking—poor ability to perceive or understand a situation from another’s point of view (Schreiter et al., 2013). Burnout was regarded as difficult to distinguish from depression (Papathanasiou, 2015), and there was significant empirical evidence of a negative relationship between empathy (both cognitive and affective empathy) and burnout of healthcare professionals (Wilkinson et al., 2017). Furthermore, a neuroimaging study demonstrated that medical professionals’ burnout was associated with reduced brain activity related to empathy (Tei et al., 2014).

On the other hand, empathy can prevent depression in healthcare professionals (Halpern, 2003; Zenasni et al., 2012; Lamothe et al., 2014). Lamothe demonstrated that the interaction of empathic processes might serve a protective role against burnout among healthcare professionals (Lamothe et al., 2014). According to Halpern, empathy helps healthcare professionals appreciate the personal meaning of patients’ words and maintains their attention on what is significant (Halpern, 2003). Such processes facilitate trust and disclosure, and can be directly therapeutic by enhancing the meaningfulness and satisfaction derived from a career in healthcare. These processes result in prevention of depression and distressing symptoms and promote the personal well-being of healthcare professionals.

A growing number of studies emphasize that daily affective states and moods directly impact on various aspects of people from mental processing (e.g., depression, anxiety) to the social information processing—the way of thinking, judgment, decision-making and interpersonal behavior on the interaction of cognitive and motivational mechanisms (Beedie et al., 2005; Martin and Clore, 2013; Desmet, 2015; Forgas, 2017). Therefore, healthcare professionals also deal with their own emotional states and traits on a daily clinical basis.

Mood and emotion are both monitoring systems that serve related, but different functions in protecting and increasing our own well-being. Although the terms of mood and emotion are used interchangeably, a clear distinction between mood and emotion has been proposed (Martin and Clore, 2013; Desmet, 2015; Forgas, 2017; Bulagang et al., 2020; Herz et al., 2020). The key difference is that mood focuses on monitoring the internal state, whereas emotion focuses on monitoring the external stimuli and environment. Some specific events such as opportunities (positive emotion) or threats (negative emotion) can evoke emotions. Because these events generally require our immediate attention, the emotion interrupts “ongoing thought and behavior” with emotion-related action tendencies that empower one to capitalize on the opportunity or neutralize the threat. By contrast, mood is not a response to external stimuli and influences to regulate the balance between one’s overall personal resources and one’s life challenges, rather than interrupting out ongoing thought and behavior.

The salutogenic theory, proposed by Antonovsky, focuses on the individual’s health-promoting resources (Antonovsky, 1979, 1987, 1996). These resources include personal physical capacities, psychological traits, knowledge, immunity, reliability of others, social support, and reward. In this theory, SOC is defined as the ability to comprehend situations in their entirety, and the capacity to use available health-promoting resources, but not as a particular personality trait or coping style (Antonovsky, 1996; Eriksson, 2022). SOC is composed of three components: comprehensibility, manageability, and meaningfulness (Moksness, 2021; Eriksson, 2022). Research has demonstrated that SOC is increased by a therapeutic approach and decreased by traumatic events (Leys et al., 2018; Mc Gee et al., 2018). A meta-analysis including an examination of informal caregivers demonstrated that SOC was an important determinant of caregiver well-being, and might protect caregivers from high levels of psychological distress and burden (del-Pino-Casado et al., 2019). Such evidence demonstrates that SOC plays a role in the prevention of depression.

When seeking to understand the relationship between empathy and SOC, it is necessary to consider the moderating role of age. According to Antonovsky (Antonovsky, 1987), younger individuals’ SOC is not yet fully developed, and is therefore more sensitive to change and disruption. So far it has been suggested that an individual’s SOC is built up during childhood and adolescence, and stabilizes around at age 30 (Antonovsky, 1987; Eriksson and Lindström, 2005; Dziuba et al., 2021). Although SOC may be more stable among people over 30 years old than among younger adults, recent studies have notably revealed that SOC at baseline rather develops after age of 30 years throughout individuals’ adult life (Feldt et al., 2007a; Nilsson et al., 2010; Dziuba et al., 2021). Furthermore, an fMRI neuroimaging study demonstrated that the neural bases of empathy change across age groups from adolescence to old age (Riva et al., 2018). Therefore, we hypothesize that age will moderate the association between individual’s empathy and self-moods through SOC mediation.

In this study, we paid more attention predominantly to a “cognitive” attribute of empathy in the context of medical settings, because a clinically-situated empathy is conceptually different from the general empathy in various social situation (Hojat et al., 2009; Michalec and Hafferty, 2021) and involves an “understanding” of the kind and quality of patients’ experiences. Cognitively defined empathy leads to personal growth, career satisfaction, and optimal clinical outcomes (Hojat et al., 2009), while developing SOC improves our ability to thrive in high-stimulus work environments with potential threats against our own well-being (Dames, 2022).

Notably, it is important to consider the difference between empathic process for other’s positive and negative affects (Hayama et al., 2008; Sawada and Hayama, 2012). Westman et al. (2013) demonstrated that empathy was associated with crossover of positive affect, but not negative affect, when stimulated by the presentation of an affective story script. Neuroimaging studies showed that neural processing of empathy might differ across the positive and negative effects of others (Balconi and Vanutelli, 2017). Moreover, positive mood states are reported to play an important role in the prevention of depressive mood (Fredrickson et al., 2008; Gruber et al., 2013; Santos et al., 2013). Unlike the known correlates of depressive mood, outcomes associated with positive mood are favorable, including friendship development (Waugh and Fredrickson, 2006), marital satisfaction (Harker and Keltner, 2001), and physical health (Richman et al., 2005).

Therefore, the aims of this study were to test the following three hypotheses:

Hypothesis 1: Empathy correlates with self-vigor or self-depression mood in healthcare professionals.

Hypothesis 2: Empathy correlates with self-vigor or self-depression mood through SOC mediation.

Hypothesis 3: Age moderates the SOC mediation on the relation between empathy and self-moods.

Firstly, we tested these hypotheses by applying one’s own positive and negative moods in healthcare professionals. Secondly, the hypotheses were tested by applying the subcategory of empathy (cognitive empathy, affective empathy for other’s negative emotions, and affective empathy for other’s positive emotions) to explore the difference between one’s own positive and negative mood among healthcare professionals.

These goals will be important to identify elements to fine-tune one’s own well-being with a clinically-situated empathy, and to provide useful indications for designing the intervention of empathy skill with SOC development among health professionals.

## Materials and Methods

### Participants

Tsuchiura Kyodo General Hospital is an 800-bed general hospital that serves as a central medical care facility for Ibaraki prefecture. Participants were recruited at the workshop portion of a hospital-wide training program “Smile Sun Project” for healthcare professionals (Takayanagi et al., 2012). We collected all data from the participants as a pre-training survey before attending the specific training program. Therefore, all participants were treatment-naïve of the training program. Healthcare professionals (*n* = 132) provided written informed consent to participate in the study. All participants were Japanese. Of these 132 potential participants, full data with no missing responses to items related to the scales used in this study were obtained for 114 participants. Of the 114 participants, 36 (31.6%) were men. Mean age was 32.8 years (SD 8.85 range = 21–60). The average age of male participants was 35.57 years (SD 13.14, range = 21–60 years), and that of female participants was 30.64 years (SD 7.31, range = 21–52 years). Participants consisted of nurses (*n* = 47; 41.2%), physical therapists (*n* = 28; 24.6%), medical doctors (*n* = 17; 14.9%), medical clerks (*n* = 8; 7.0%), radiologic technologists (*n* = 5; 4.4%), pharmacists (*n* = 4; 3.5%), medical technologists (*n* = 2; 1.8%), medical social workers (*n* = 2; 1.8%), and a clinical psychologist (*n* = 1; 0.9%).

### Measures

#### Empathy Process Scale

Empathy was evaluated according to the Empathy Process Scale (EPS), which was recently developed by Hayama et al. (2008) based on the Interpersonal Reactivity Index proposed by Davis (1983). This Japanese 30-item questionnaire was designed with six sub-dimension scores to assess both the cognitive and emotional aspects of empathy (Sawada and Hayama, 2012; Ohnishi et al., 2017). This scale focuses on more detailed emotional aspects than that developed by Davis. The emotional aspects of empathy include “Sharing positive emotions with others,” “Good feeling for others’ positive emotions,” “Sharing negative emotions with others,” and “Sympathy for others’ negative emotions.” The cognitive aspects of empathy include “Perspective taking” and “Sensibility about others’ emotions.” The self-reported responses were provided on a 5-point Likert-type scale with the anchors of 1 = Strongly disagree and 5 = Strongly agree. All six subscales consisted of five items with scores ranging from 5 to 25. In this study we defined three categories of empathy scale: (1) the cognitive aspects of empathy as EPS-C (Empathy Process Scale for Cognition) representing “Perspective taking” and “Sensibility about others’ emotions”; (2) empathy for other’s positive affects as EPS-P (Empathy Process Scale for other’s Positive affect) representing “Sharing positive emotions with others” and “Good feeling for others’ positive emotions”; (3) empathy for other’s negative affects as EPS-N (Empathy Process Scale for other’s Negative affects) representing “Sharing negative emotions with others,” and “Sympathy for others’ negative emotions.” The internal consistency of the Empathy Process Scale used in this study was demonstrated with a Cronbach’s α of 0.814.

#### Sense of Coherence

Permission to use Sense of coherence (SOC) questionnaire has been granted through the Society for Theory and Research on Salutogenesis^1^. SOC was assessed with a 13-item abbreviated Japanese version of the 29-item Antonovsky’s Orientation to Life Questionnaire (Tsuno et al., 2017). Respondents were asked to rate their level of agreement with each of the items on a 7-point scale. SOC-13 measures the three key aspects of SOC: meaningfulness (4 items), comprehensibility (5 items), and manageability (4 items). The total SOC score is the sum of these items and ranges from 13 to 91. Its sub-dimensions range from 5 to 35 (comprehensibility) and 4–28 (manageability and meaningfulness). The internal consistency of the SOC-13 scale used in this study was demonstrated with a Cronbach’s α of 0.848.

#### Profile of Mood States

Situational mood was assessed *via* the Profile of Mood States (POMS) short Japanese version (Yokoyama et al., 1990; McNair et al., 1992; Yokoyama, 1994). This short version consists of 30 items that evaluate six emotional subscales: tension-anxiety (5 items), depression-dejection (5 items), anger-hostility (5 items), vigor (5 items), fatigue (5 items), and confusion (5 items). Participants were asked to assess their mood during the past week on a 5-point scale ranging from 0 (never) to 4 (very much) for each item. POMS-Depression (POMS-D) assessed depressive mood. POMS-Vigor (POMS-V) assessed vigorous mood. T-scores were used to assess participants’ mood states (McNair et al., 1992). A T-score conversion table was used to convert the raw score to the T-score. The average T-score for Japanese individuals is 50 points (Yokoyama, 1994). The internal consistency of the POMS scale used in this study was demonstrated with a Cronbach’s α of 0.831.

#### Statistical Analysis

Analyses were performed using SPSS, version 25 (SPSS Inc., Chicago) and PROCESS 3.2 (Hayes, 2018); with alpha levels set at *p* < 0.05. A Kolmogorov–Smirnov test suggested that all variables except “sharing negative affects with others (*p* = 0.061)” in EPS were not normally distributed. Therefore, Spearman’s rank correlation coefficient (*r*) was used for bivariate correlation analysis to examine the intercorrelation among age, sex, depression mood state, vigor mood state, SOC, EPS, and subscale as univariate analysis. Regarding the violation of normal distribution in regression analysis, previous studies involving simulations indicated that linear regression analyses of large samples are valid for any distribution, and are only invalid when the sample size is quite small (Lumley et al., 2002; Hayes, 2018). We therefore analyzed raw data this study. Levene’s test suggested heteroscedasticity, indicating the potential for results to be distorted. However, research involving simulations suggests that minor violations of the homoscedasticity assumption are not a major cause for concern (Hayes, 2018). Therefore, we chose the HC3 heteroscedasticity-consistent standard error estimator, one of the options within the PROCESS macro in SPSS (Hayes and Cai, 2007).

**FIGURE 1:**
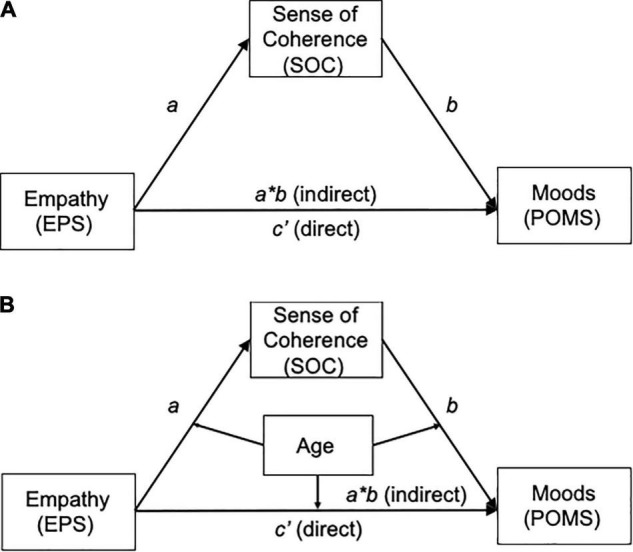
**(A)** Schematic model of sense of coherence (SOC) as a mediator between empathy (EPS) and self-moods (POMS): the proposed Model 4 adapted from Hayes (2018). X, independent variable; Y, dependent variable; M, mediator. The path *a*, X (EPS) to M (SOC); the path *b*, from M (SOC) to Y (POMS). The path *c’* means the “direct” effect of X (EPS) on Y (POMS) and the path a*b means “indirect” effect of X on Y through M. The total effect of X (EPS) on Y (POMS) means the sum of the direct effect (*c’*) and the indirect effect (a*b). EPS, empathy process scale; SOC, sense of coherence; POMS, profile of mood state. **(B)** Schematic model of age as a moderator of the mediation model: the proposed Model 59 adapted from Hayes (2018). X, independent variable; Y, dependent variable; M, mediator; Mo, moderator. The path *a*, X (EPS) to M (SOC); the path *b*, from M (SOC) to Y (POMS). The path *c’* means the “direct” effect of X (EPS) on Y (POMS) and the path a*b means “indirect” effect of X on Y through M. The total effect of X (EPS) on Y (POMS) means the sum of the direct effect (*c’*) and the indirect effect (a*b). All pathways are moderated by Mo (age). EPS; Empathy process scale; SOC: Sense of coherence; POMS: Profile of mood state.

Mediation analysis: Figure 1A illustrates the hypothesized mediation model whereby empathy (EPS, and EPS subcategories) affected self-moods (POMS-V and POMS-D) *via* SOC. These mediation analyses were conducted using Model 4 of the PROCESS macro (Hayes and Cai, 2007; Hayes, 2018). This approach tests the regression coefficients for the effects of an independent variable (EPS: empathy) on a dependent variable (POMS: self-mood) were calculated in Step 1 as total effect: the sum of the direct (*c’*) and indirect (a*b) effects, an independent variable on a mediator (SOC) in Step 2 (path *a*), and a mediator on a dependent variable controlling for the independent variable in Step 3 (path *b* and the “direct” effect path *c’*). Then, the sizes of the indirect effects of the mediators were estimated using a bias-corrected bootstrapping method with 5,000 replications, resulting in bootstrap 95% confidence intervals (CIs). An indirect mediation effect was deemed significant when the bootstrap 95% CI excluded zero (equivalent to 0.05 levels). These mediation analyses were conducted using Model 4 of the PROCESS macro (Hayes and Cai, 2007; Hayes, 2018).

Moderated mediation analysis: Figure 1B illustrates the hypothesized moderated mediation model whereby empathy (EPS, and EPS subcategories) affected mood (POMS-V and POMS-D) *via* SOC, and each association was moderated by age. Model 59 of the PROCESS macro was used for the moderation analysis (Steps 4 and 5) to test whether the indirect paths were moderated by age (Hayes and Cai, 2007). We utilized the simple slopes method to visually represent the moderation when significant interaction associations were detected. These interactions were illustrated by depicting the regression lines of the relation at low (mean – 1 SD), medium (mean), and high scores (mean + 1 SD) of the moderator variable (age). Next, we conducted a *post hoc* analysis using the Johnson–Neyman technique (Preacher et al., 2006; Hayes, 2018) to identify the regions within the range of the moderator variable where the association between variables on the dependent variable was or was not statistically significant. We also analyzed the sub-categories of empathy (EPS-C, EPS-N, and EPS-P). Likewise, moderated mediation indexes were deemed significant if the 95% CI excluded zero (equivalent to 0.05 levels).

## Results

### Descriptive Statistics

Descriptive statistics and correlations for the measured variables are presented in Table 1. Results indicated that empathy score (EPS), SOC, and self-vigor mood (POMS-V) were positively interrelated. In addition, both empathy (EPS) and SOC were negatively associated with self-depression mood (POMS-D). We observed significant associations with each measure of three empathy (EPS) sub-categories: the cognitive aspects of empathy (EPS-C), empathy for other’s negative affects (EPS-N), and empathy for other’s positive affects (EPS-P). SOC was positively associated with both the cognitive aspects of empathy (EPS-C) and empathy for other’s positive affects (EPS-P). Self-depression mood (POMS-D) was negatively associated with empathy for other’s positive affects (EPS-P). Self-vigor mood (POMS-V) was positively associated with both empathy for other’s negative affects (EPS-N) and empathy for other’s positive affects (EPS-P).

**TABLE 1:** Means, standard deviations and correlations among the study variables.

	Age	SOC	POMS-D	POMS-V	EPS	EPS-C	EPS-N	EPS-P
Age								
SOC	0.13							
POMS-D	–0.08	−0.60**						
POMS-V	0.11	0.37**	–0.16					
EPS	–0.05	0.30**	−0.21*	0.31**				
EPS-C	–0.13	0.22*	–0.14	0.18	0.84**			
EPS-N	0.08	0.17	–0.10	0.31**	0.82**	0.53**		
EPS-P	–0.05	0.39**	−0.34**	0.32**	0.84**	0.56**	0.58**	
Means	32.83	54.84	53.91	46.82	113.41	38.12	36.39	38.89
SD	8.86	12.68	12.49	8.81	14.82	5.68	5.52	6.13

*SOC, sense of coherence; POMS-D, profile of mood state depression-dejection; POMS-V, profile of mood state vigor; EPS, empathy process scale; EPS-C, empathy process scale for cognition; EPS-N, empathy process scale for other’s negative affects; EPS-P, empathy process scale for other’s positive affects; SD, standard deviation.*

**p < 0.01, *p < 0.05, N = 114.

### Empathy on Self-Vigor Mood Through Sense of Coherence

#### Mediation Analysis

**FIGURE 2:**
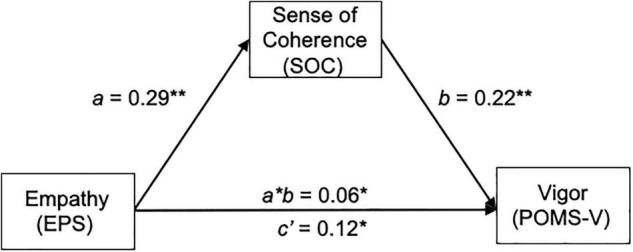
Mediation effect of SOC on the relationship between empathy and self-vigor mood (POMS-V). EPS, empathy; SOC, sense of coherence; POMS-V, profile of mood state vigor.

Figure 2 and Table 2 present the mediation effect of SOC on the association between empathy (EPS) and self-vigor mood (POMS-V). As shown in Supplementary Table 1, empathy (EPS) was positively associated with self-vigor mood (POMS-V) (Step 1: total effect *B* = 0.19, SE = 0.0502, *t* = 3.764, *p* = 0.0003) and SOC (Step 2: path *a B* = 0.29, SE = 0.0687, *t* = 4.2634, *p* < 0.001). Furthermore, both empathy (EPS) and SOC positively predicted self-vigor mood (POMS-V) with significance (Step 3: the direct effect c’ *B* = 0.1245, SE = 0.0519, *t* = 2.398, *p* = 0.0182, and the path *b B* = 0.2202, SE = 0.067, *t* = 3.3006, *p* = 0.0013, respectively). Analysis from bias-corrected bootstrapping with 5,000 samples confirmed a significant positive-indirect effect of empathy (EPS) on the self-vigor mood (POMS-V) through SOC [the indirect effect a*b B = 0.06, BootSE = 0.03, 95%CI (0.02, 0.12)], as shown in Table 2.

**TABLE 2:** Indirect effect of SOC on the association between empathy (EPS) and depressive (POMS-D) and positive mood (POMS-V).

		B	BootSE	BootLLCI	BootULCI
POMS-V	EPS	0.06	0.03	0.02	0.12
	EPS-C	0.15	0.07	0.03	0.29
	EPS-N	0.11	0.06	0.01	0.23
	EPS-P	0.19	0.07	0.05	0.33
POMS-D	EPS	–0.22	0.06	–0.34	–0.11
	EPS-C	–0.46	0.17	–0.79	–0.12
	EPS-N	–0.33	0.14	–0.62	–0.05
	EPS-P	–0.59	0.13	–0.86	–0.34

*B, regression coefficient; SE, standard error; LLCI, lower limit of confidential interval; ULCI, upper limit of confidential interval.*

*When the bootstrap 95% CI did not include zero, the indirect association was taken to be significant at the 0.05 level.*

*POMS-D, profile of mood state depression-dejection; POMS-V, profile of mood state vigor; EPS, empathy process scale; EPS-C, empathy process scale for cognition; EPS-N, empathy process scale for other’s negative affects; EPS-P, empathy process scale for other’s positive affects.*

#### Moderated Mediation Analysis

We invested whether age moderated the mediation effect of SOC on the association between empathy (EPS) and self-vigor mood (POMS-V). SOC was predicted neither by EPS (Step 4: *B* = –0.1538, SE = 0.2789, *t* = –0.5513, *p* = 0.5825, Supplementary Table 1) nor the interaction effect of empathy (EPS) and age (Step 4: *B* = 0.0129, SE = 0.0075, *t* = –0.5513, *p* = 0.0915, Supplementary Table 1). Notably, the self-vigor mood (POMS-V) in healthcare professionals was significantly predicted by the interaction effect of SOC and age (Step 5, *B* = 0.013, SE = 0.0064, *t* = 2.0179, *p* = 0.0461, Supplementary Table 1).

Result of the Johnson–Neyman method indicated that there was a significant positive association between SOC and POMS-V when age exceeded 31.48, but there was no significant association between them in ages under 31.48. Regarding the conditional indirect effect of EPS on POMS-V thorough SOC, bootstrap results indicated no significant indirect effect at mean age – 1 SD “23.97” [*B* = 0.009, Boot SE 0.020, 95%CI (−0.031, 0.055)], and a significant indirect effect of EPS on POMS-V through SOC score at mean age “32.83” [*B* = 0.046, Boot SE 0.024, 95% CI (0.004, 0.096)] and at mean age + 1 SD “41.69” [*B* = 0.110, Boot SE 0.039, 95% CI (0.037, 0.193)].

### Empathy on Self-Depression Mood Through Sense of Coherence

#### Mediation Analysis

**FIGURE 3:**
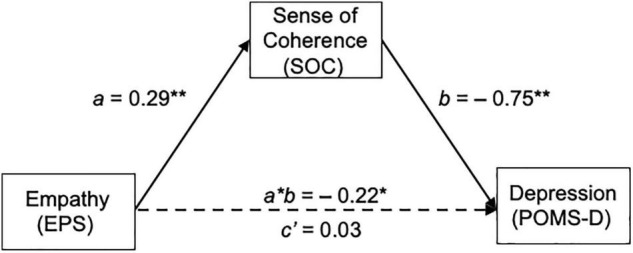
Mediation effect of SOC on the relationship between empathy and self-depression mood (POMS-D). EPS, empathy; SOC, sense of coherence; POMS-D, profile of mood state depression-dejection.

Figure 3 and Table 2 present the mediation effect of SOC on the association between empathy (EPS) and self-depression mood (POMS-D). As shown in Supplementary Table 2, empathy (EPS) was significantly associated with self-depression mood (POMS-D) (Step 1: total effect *B* = −0.19, SE = 0.078, *t* = −2.4665, *p* = 0.0152) and SOC (Step 2: path *a B* = 0.29, SE = 0.0687, *t* = 4.2634, *p* < 0.001). Furthermore, SOC negatively predicted self-depression mood (POMS-D) with significance (Step 3: path *b B* = −0.75, SE = 0.0815, *t* = −9.212 *p* < 0.001), whereas the direct effect of empathy (EPS) on self-depression mood (POMS-D) was non-significant (Step 3: the direct effect *c’ B* = 0.03, SE = 0.0634, *p* = 0.4352), indicating full mediation. Analysis from bias-corrected bootstrapping with 5,000 samples confirmed significant negative-indirect effects of empathy (EPS) on self-depression mood (POMS-D) through SOC [the indirect effect a*b B = –0.220, BootSE = 0.06, 95%CI (–0.34, –0.11)] in Table 2.

#### Moderated Mediation Analysis

We invested whether age moderated the mediation effect of SOC on the association between empathy (EPS) and self-depression mood (POMS-D). As shown in Supplementary Table 2, there was no significant interaction in [empathy (EPS) × age] on SOC or (SOC × age) on the POMS-D.

### Sub-Category of Empathy on Depressive Mood Through Sense of Coherence

#### Mediation Analysis

In this study we defined three sub-categories of empathy scale as the cognitive aspects of empathy (EPS-C), empathy for other’s negative affects (EPS-N), and empathy for other’s positive affects (EPS-P). Therefore, we examined how each sub-category of empathy (EPS-C, EPS-N, and EPS-P) may predict depressive mood in healthcare professionals mediated by SOC. EPS-P was negatively associated with self-depression mood (POMS-D) (step 1: total effect in Supplementary Table 4), but neither EPS-C nor EPS-N was associated with POMS-D (step 1: total effect in Supplementary Tables 3, 5). All three sub-categories of EPS were positively associated with SOC (Step 2: path *a*, in Supplementary Tables 3–5). The association between SOC and self-depression mood (POMS-D) was significant after adjusting all EPS three sub-categories (Step 3: path *b* in Supplementary Tables 3–5). Analysis from bias-corrected bootstrapping with 5,000 samples confirmed a negative indirect effect of all EPS three sub-categories on both moods through SOC with significant in Table 2.

#### Moderated Mediation Analysis

Among three sub-categories of empathy, there was a significant interaction of (EPS-N × age) on SOC (step 4 and 5 in Supplementary Table 5). The result of the Johnson–Neyman method indicated that there was a significant positive association between SOC and self-depression mood (POMS-D) in ages greater than 33.0 years, while there was no significant association between them ages less than 33.0. Regarding conditional indirect effects of EPS-N on POMS-D mediated by SOC, there was significant indirect effect at mean age “32.83” [*B* = −0.277, BootSE 0.141, 95%CI (−0.566, −0.0103)] and at mean age + 1 SD “41.69” [*B* = −0.626, BootSE 0.0197, 95%CI (−1.068, −0.291)], but non-significant effect of EPS-N on POMS-D thorough SOC at mean age – 1 SD “23.97” [*B* = 0.125, BootSE 0.226, 95%CI (−0.307, 0.597)].

## Discussion

We tested the moderated mediation model of SOC between empathy and self-mood states among healthcare professionals in a general hospital. Firstly, the current study identified that higher levels of empathy were linked to higher SOC, which was in turn positively related to self-vigor mood of healthcare professionals. This SOC-mediated relation was moderated by age. The moderated mediation model significantly explained approximately 30% (*R*^2^ = 0.29 for the SOC mediation and *R*^2^ = 0.35 for the SOC × Age moderation) of self-vigor mood. These results supported our three moderated-mediational hypotheses. Secondly, this SOC-mediated pattern was also observed regarding self-depression mood reduction among healthcare professionals. By contrast, higher empathy was linked to higher SOC, which was then related to lower levels of self-depression mood. This model significantly explained approximately 47% (*R*^2^ = 0.47 for the SOC mediation) of self-depression mood, although a moderative effect of age did not occur through this mediation. These results supported our mediational hypotheses 1 and 2, but not moderation hypothesis 3. Thirdly, regarding the empathy for other’s negative affections (EPS-N: the subcategories of empathy), age might function as a moderator on the SOC-mediated association between ESP-N and self-depression mood. Collectively, empathy in healthcare professionals might promote self-vigor and might serve a preventative role against self-depression mood mediated through SOC. Some parts of the process depended on age.

Interestingly, the boundary between significant and insignificant associations was correlated with the age of around 30 years—the same as has been identified as the age at which development of an individual’s SOC is more stable. The current results notably suggest that SOC may not interfere with self-vigor mood until SOC is fully established. In other words, it might be difficult for younger healthcare professionals who have less clinical experience and are less skilled in using empathy as a resource in their healthcare context, which makes it difficult to increase self-vigor mood from SOC. Age may co-vary with the accumulation of various life experience, including clinical experience. Therefore, it might be speculated that developing SOC *via* empathy for other’s affections in medical care is related to an experience accumulated with age, and in turn regulates to increase self-vigor mood.

Sense of coherence is an individual’s way of thinking, being, and acting on a one’s view of life and ability to respond to stressful environments with promoting health and well-being (Krok, 2020; Eriksson, 2022). Developing SOC will lead people to identify one’s internal and external resources during one’s life stage. Further, thinking and understanding why and how resources work will allow one for more flexible use of resources and define what goals should be pursued. Therefore, SOC is closely related with the individual’s perception of one’s ability to behave through cognitive (*Comprehensibility* of SOC) and motivational (*Meaningfulness* of SOC) processes (Moksness, 2021; Shorey and Lopez, 2021). These concepts of SOC seem to be parallel with the concept of mindfulness, that has currently been proposed as another concept for the implementation of healthcare professionals’ well-being (Grevenstein et al., 2018; Dames, 2022). Both focus on the meta-cognitive development, emphasizing a sharpened awareness of present phenomena, free of insecure attachments, constant practice of moving from subjective thinking mind to the objective sensing mind. Meta-cognition is thinking about one’s thinking and having a meta-cognition habit of mind is essential for healthcare professionals. Developing skills in meta-cognition may also benefit for SOC growing among health professionals (Eichbaum, 2014; Medina et al., 2017; Versteeg et al., 2021).

Similarly, empathy promotes comprehension of the personal meanings of patients’ distress in the process of providing healthcare (Halpern, 2003). This understanding may promote high quality of care as well as result in improved self-management among healthcare professionals. Empathy may make providing healthcare more meaningful and enhance the provider’s satisfaction. In turn, this meaningfulness in their daily work may promote self-vigor mood states.

We should discuss the potential alternative relationship among empathy, SOC, and self-vigor mood or self-depression mood, because causal relationships between these factors could not be determined due to the cross-sectional nature of the study design. First, previous reports suggested that SOC was correlated with the Big Five personality traits, and at least some components of SOC might reflect the interplay of these fundamental Big Five traits as the predictor of psychological well-being (Feldt et al., 2007b; Hochwälder, 2012; Kase et al., 2018; Leys et al., 2018; Mc Gee et al., 2018). However, based on Antonovsky’s salutogenic theory, SOC concept has been emphasized to focus on entire person and salutary factors rather than a personality trait or a coping strategy (Antonovsky, 1996). The accumulating evidences indicated that SOC independently predicted mental health related to factors other than the Big Five personality traits (Grevenstein and Bluemke, 2015; Grevenstein et al., 2018). Second, it is also possible that SOC is associated with both self-vigor mood and self-depression mood mediated through empathy. However, the findings of the current study do not seem to support such mediation models due to the absence of significant indirect association in such mediation models (data not shown). Third, it is plausible that self-depression mood may affect empathy. Schreiter et al. (2013) proposed that depression promoted empathic stress, which was evoked by imagining how someone would react when presented with another person’s stressful situation, resulting in decreased empathy. Cognitive distortion, such an interpretation bias of depression, might affect cognitive empathy (Wisco and Nolen-Hoeksema, 2010). On the other hand, positive emotions may also affect empathy. Waugh and Fredrickson (2006) demonstrated that positive affects predicted a complex understanding of one’s roommate, which carried implications for the role of positive affects in the formation of new relationships between college students. These processes suggested that positive affects promote empathy. Although we measured trait empathy, not context-induced state empathy, we cannot dismiss the possibility that these possible associations of self-vigor mood and self-depression mood affected the measurement of empathy in the current study.

The results of the present study implied differences between self-vigor mood and self-depression mood through empathy. Empathy for others’ positive emotions (EPS-P) affected the self-depression mood of healthcare professionals of any age through SOC. Meanwhile, we observed no significant effect of empathy for others’ negative emotions (EPS-N) on the self-depression mood of younger healthcare professionals (<24 years old). Empathy was suggested to be associated with a crossover of positive affects, but not negative affects, in a study involving participants who were young army trainees with an age of 18 or 19 years (Westman et al., 2013). There are intervention programs for healthcare professionals to improve empathic behaviors, and a systematic review suggested that they bring about significant improvement, although the type of intervention that would be effective remains unclear (Kiosses et al., 2016).

This study thereby makes an original contribution to the literature: as an empathy-organizing model we integrated the individual empathy skill and SOC growth with age to fine-tune one’s own emotional regulation among healthcare professionals, which may increase vigor mood and dampens depression mood for leading to beneficial consequences of well-being states.

### Limitations

This study had several limitations beyond those already mentioned. First, the moderation analysis was based on the assumption of the temporal precedence of the moderators of empathy and sense of coherence (and mood state) (Kraemer et al., 2008). Although the significant moderating association of age is consistent with this view, our correlational methodology does not rule out a different temporal sequence. Second, all of the information was self-reported, and non-differential misclassification may be inevitable and could attenuate the observed associations. Third, all participants were Japanese, and were of varied vocations and levels of training (i.e., medical doctor, nurse, and others), rather than members of a single medical discipline. Additionally, results may be distorted by the sampling method. Restricting the sample to healthcare professionals from a single institute in one Japanese prefecture may pose a risk to the validity of the obtained data by leaving open the possibility that the participants are not representative of healthcare professionals more generally. Finally, residual confounding by uncontrolled or unmeasured factors may have distorted the results of the analysis.

## Conclusion

The results of the current study suggested that empathy in healthcare professionals might promote self-vigor mood, and might serve a protective role against self-depression mood mediated through SOC. However, among younger healthcare professionals, SOC might not mediate the association between empathy and self-vigor mood, and the association between empathy for other’s negative affects and self-vigor/depression moods.

Collectively, the current study indicated the importance of individual differences such as the growth level of SOC among healthcare professionals. It can potentially contribute to the intervention design and implementation to regulate the empathy skills, especially for keeping psychological well-being with considering the individual age-related SOC developing. Further longitudinal studies, including examinations of interventions for the clinically-situated empathy and SOC development, are needed to elucidate causality of the association among empathy, SOC, and self-vigor/depression moods.

## Data Availability Statement

The original contributions presented in the study are included in the article/Supplementary Material, further inquiries can be directed to the corresponding author/s.

## Ethics Statement

The studies involving human participants were reviewed and approved by Tsuchiura Kyodo General Hospital. The patients/participants provided their written informed consent to participate in this study.

## Author Contributions

MH, SS, TO, YS, HF, KT, KM, and JO contributed to conception and design of the study. SS organized the database. EY and DH performed the statistical analysis. JO and EY wrote the first draft of the manuscript. MH wrote sections of the manuscript. All authors contributed to manuscript revision, read, and approved the submitted version.

## Conflict of Interest

The authors declare that the research was conducted in the absence of any commercial or financial relationships that could be construed as a potential conflict of interest.

## Publisher’s Note

All claims expressed in this article are solely those of the authors and do not necessarily represent those of their affiliated organizations, or those of the publisher, the editors and the reviewers. Any product that may be evaluated in this article, or claim that may be made by its manufacturer, is not guaranteed or endorsed by the publisher.

## Footnotes

1
https://www.stars-society.org
