# Supplementary material for: Sense of Coherence as a Mediator in the Association Between Empathy and Moods in Healthcare Professionals: The Moderating Effect of Age
Source: Front Psychol. 2022 Apr 25;13:847381. doi: 10.3389/fpsyg.2022.847381 (PMC9083206; doi:10.3389/fpsyg.2022.847381)
Supplement: Supplementary file 1 [file Data_Sheet_1.docx]

Supplemental Table 1. Result of moderated mediation analysis: indirect effect of empathy on positive mood through sense of coherence moderated by age

|  | **Mediation analysis** | | | | | | | |  | **Moderated Mediation analysis** | | | | |
| --- | --- | --- | --- | --- | --- | --- | --- | --- | --- | --- | --- | --- | --- | --- |
| **Dependent variable** | POMS-V | |  | SOC | |  | POMS-V | |  | SOC | |  | POMS-V | |
|  | Step 1 | |  | Step 2 | |  | Step 3 | |  | Step 4 | |  | Step 5 | |
| **Independent variable** | B | SE |  | B | SE |  | B | SE |  | B | SE |  | B | SE |
| Constant | 19.78 | 7.20 |  | 8.93 | 8.83 |  | 17.81** | 6.60 |  | 61.20 | 32.69 |  | 73.56** | 21.59 |
| EPS | 0.19** | 0.06 |  | 0.29** | 0.07 |  | 0.12* | 0.05 |  | – 0.15 | 0.28 |  | – 0.11 | 0.21 |
| SOC |  |  |  |  |  |  | 0.22** | 0.07 |  |  |  |  | – 0.25 | 0.24 |
| Age | 0.12 | 0.10 |  | 0.31** | 0.11 |  | 0.05 | 0.09 |  | **–** 1.20 | 0.89 |  | – 1.49 | 0.56 |
| **Interaction items** |  |  |  |  |  |  |  |  |  |  |  |  |  |  |
| EPS × Age |  |  |  |  |  |  |  |  |  | 0.01 | 0.01 |  | 0.01 | 0.01 |
| SOC × Age |  |  |  |  |  |  |  |  |  |  |  |  | 0.01* | 0.01 |
| R^2^ | 0.22 | |  | 0.29 | |  | 0.29 | |  | 0.31 | |  | 0.35 | |

B: Regression coefficient; SE: Standard Error; EPS: Empathy process scale; SOC: Sense of coherence; POMS- V: Profile of mood state Vigor.

***p* < *0.01*, **p* < *0.05*.

Supplemental Table 2. Result of moderated mediation analysis: indirect effect of empathy on depressive mood through sense of coherence moderated by age

|  | **Mediation analysis** | | | | | | | |  | **Moderated Mediation analysis** | | | | |
| --- | --- | --- | --- | --- | --- | --- | --- | --- | --- | --- | --- | --- | --- | --- |
| **Dependent variable** | POMS-D | |  | SOC | |  | POMS-D | |  | SOC | |  | POMS-D | |
|  | Step 1 | |  | Step 2 | |  | Step 3 | |  | Step 4 | |  | Step 5 | |
| **Independent variable** | B | SE |  | B | SE |  | B | SE |  | B | SE |  | B | SE |
| Constant | 78.44 | 9.20 |  | 8.93 | 8.83 |  | 85.14** | 6.80 |  | 61.20 | 32.69 |  | 96** | 23.81 |
| EPS | **–** 0.19* | 0.08 |  | 0.29** | 0.07 |  | 0.03 | 0.06 |  | **–** 0.15 | 0.28 |  | 0.09 | 0.21 |
| SOC |  |  |  |  |  |  | **–** 0.75** | 0.08 |  |  |  |  | **–** 1.04** | 0.25 |
| Age | – 0.07 | 0.12 |  | 0.31** | 0.11 |  | 0.16 | 0.10 |  | **–** 1.20 | 0.89 |  | **–** 0.12 | 0.71 |
| **Interaction items** |  |  |  |  |  |  |  |  |  |  |  |  |  |  |
| EPS × Age |  |  |  |  |  |  |  |  |  | 0.01 | 0.01 |  | 0.00 | 0.01 |
| SOC × Age |  |  |  |  |  |  |  |  |  |  |  |  | 0.01 | 0.01 |
| R^2^ | 0.06 | |  | 0.29 | |  | 0.47 | |  | 0.31 | |  | 0.48 | |

B: Regression coefficient; SE: Standard Error; EPS: Empathy process scale; SOC: Sense of coherence; POMS-D: Profile of mood state Depression-Dejection.

***p* < *0.01*, **p* < *0.05*.

Supplemental Table 3. Result of moderated mediation analysis: indirect effect of cognitive empathy on depressive mood through sense of coherence　moderated by age

|  | **Mediation analysis** | | | | | | | |  | **Moderated mediation** | | | | |
| --- | --- | --- | --- | --- | --- | --- | --- | --- | --- | --- | --- | --- | --- | --- |
| **Dependent variable** | POMS-D | |  | SOC | |  | POMS-D | |  | SOC | |  | POMS-D | |
|  | Step 1 | |  | Step 2 | |  | Step 3 | |  | Step 4 | |  | Step 5 | |
| **Independent variable** | B | SE |  | B | SE |  | B | SE |  | B | SE |  | B | SE |
| Constant | 64.44** | 10.55 |  | 18.23 | 10.08 |  | 78.58** | 6.59 |  | 61.37 | 30.15 |  | 82.28 | 21.64 |
| EPS-C | – 0.19 | 0.25 |  | 0.60* | 0.23 |  | 0.27 | 0.18 |  | – 0.51 | 0.78 |  | 0.62 | 0.66 |
| SOC |  |  |  |  |  |  | – 0.78** | 0.08 |  |  |  |  | – 1.06** | 0.28 |
| Age | – 0.09 | 0.12 |  | 0.35** | 0.11 |  | 0.18 | 0.11 |  | – 0.91 | 0.82 |  | 0.11 | 0.67 |
| **Interaction items** |  |  |  |  |  |  |  |  |  |  |  |  |  |  |
| EPS-C × Age |  |  |  |  |  |  |  |  |  | 0.03 | 0.02 |  | – 0.01 | 0.02 |
| SOC × Age |  |  |  |  |  |  |  |  |  |  |  |  | 0.01 | 0.01 |
| R^2^ |  | |  | 0.24 | |  | 0.48 | |  | 0.26 | |  | 0.48 | |

B: Regression coefficient; SE: Standard Error; EPS-C: Empathy process scale for Cognition; SOC: Sense of coherence; POMS-D: Profile of mood state Depression-Dejection.

***p* < *0.01*, **p* < *0.05*.

Supplemental Table 4. Result of moderated mediation analysis: indirect effect of empathy for other's positive affect on depressive mood through sense of coherence moderated by age

|  | **Mediation analysis** | | | | | | | |  | **Moderated mediation** | | | | | | | |
| --- | --- | --- | --- | --- | --- | --- | --- | --- | --- | --- | --- | --- | --- | --- | --- | --- | --- |
| **Dependent variable** | POMS-D | |  | SOC | |  | POMS-D | |  | SOC | | |  | | POMS-D | | |
|  | Step 1 | |  | Step 2 | |  | Step 3 | |  | Step 4 | | |  | | Step 5 | | |
| **Independent variable** | B | SE |  | B | SE |  | B | SE |  | B | SE |  | | B | | SE |  |
| Constant | 85.91** | 6.95 |  | 9.35 | 7.12 |  | 92.42** | 5.41 |  | 26.12 | 29.92 |  | | 103.60** | | 17.62 |  |
| EPS-P | – 0.76** | 0.16 |  | 0.85** | 0.16 |  | – 0.17 | 0.15 |  | 0.43 | 0.73 |  | | 0.05 | | 0.37 |  |
| SOC |  |  |  |  |  |  | – 0.70** | 0.09 |  |  |  |  | | – 1.03 | | 0.23 |  |
| Age | – 0.07 | 0.11 |  | 0.31** | 0.12 |  | 0.15 | 0.10 |  | – 0.17 | 0.89 |  | | – 0.16 | | 0.52 |  |
| **Interaction items** |  |  |  |  |  |  |  |  |  |  |  |  | |  | |  |  |
| EPS-P × Age |  |  |  |  |  |  |  |  |  | 0.01 | 0.02 |  | | – 0.01 | | 0.01 |  |
| SOC × Age |  | ­ |  |  |  |  |  |  |  |  |  |  | | 0.01 | | 0.01 |  |
| R^2^ |  | |  | 0.34 | |  | 0.48 | |  | 0.34 | | |  | | 0.48 | | |

B: Regression coefficient; SE: Standard Error; EPS-P: Empathy process scale for other’s Positive affects; SOC: Sense of coherence; POMS-D: Profile of mood state Depression-Dejection.

***p* < *0.01*, **p* < *0.05*.

Supplemental Table 5. Result of moderated mediation analysis: indirect effect of empathy for other's positive affect on depressive mood through sense of coherence moderated by age

|  | **Mediation analysis** | | | | | | | |  | **Moderated Mediation analysis** | | | | |
| --- | --- | --- | --- | --- | --- | --- | --- | --- | --- | --- | --- | --- | --- | --- |
| **Dependent variable** | POMS-D | |  | SOC | |  | POMS-D | |  | SOC | |  | POMS-D | |
|  | Step 1 | |  | Step 2 | |  | Step 3 | |  | Step 4 | |  | Step 5 | |
| **Independent variable** | B | SE |  | B | SE |  | B | SE |  | B | SE |  | B | SE |
| Constant | 65.62 | 8.25 |  | 26.38** | 7.84 |  | 85.29** | 6.60 |  | 100.88 | 26.49 |  | 112.18** | 28.22 |
| EPS-N | **–** 0.25 | 0.23 |  | 0.45* | 0.19 |  | 0.08 | 0.17 |  | **–** 1.53* | 0.70 |  | **–** 0.28 | 0.70 |
| SOC |  |  |  |  |  |  | **–** 0.75** | 0.08 |  |  |  |  | **–** 0.97** | 0.22 |
| Age | **–** 0.06 | 0.13 |  | 0.29* | 0.13 |  | 0.15 | 0.10 |  | **–** 1.89** | 0.75 |  | **–** 0.59 | 0.86 |
| **Interaction items** |  |  |  |  |  |  |  |  |  |  |  |  |  |  |
| EPS-N × Age |  |  |  |  |  |  |  |  |  | 0.06** | 0.02 |  | 0.01 | 0.02 |
| SOC × Age |  |  |  |  |  |  |  |  |  |  |  |  | 0.01 | 0.01 |
| R^2^ | 0.21 | |  | 0.21 | |  | 0.47 | |  | 0.26 | |  | 0.48 | |

B: Regression coefficient; SE: Standard Error; EPS-N: Empathy process scale for other’s Negative affects; SOC: Sense of coherence; POMS-D: Profile of mood state Depression-Dejection.

***p* < *0.01*, **p* < *0.05*.

Supplemental Table 6. Result of moderated mediation analysis: indirect effect of cognitive empathy on positive mood through sense of coherence moderated by age

|  | **Mediation analysis** | | | | | | | |  | **Moderated mediation** | | | | |
| --- | --- | --- | --- | --- | --- | --- | --- | --- | --- | --- | --- | --- | --- | --- |
| **Dependent variable** | POMS-V | |  | SOC | |  | POMS-V | |  | SOC | |  | POMS-V | |
|  | Step 1 | |  | Step 2 | |  | Step 3 | |  | Step 4 | |  | Step 5 | |
| **Independent variable** | B | SE |  | B | SE |  | B | SE |  | B | SE |  | B | SE |
| Constant | 27.84** | 7.32 |  | 18.23 | 10.08 |  | 23.20** | 6.43 |  | 61.37 | 30.15 |  | 72.33** | 21.23 |
| EPS-C | 0.33* | 0.16 |  | 0.60* | 0.23 |  | 0.18 | 0.14 |  | – 0.51 | 0.78 |  | – 0.23 | 0.62 |
| SOC |  |  |  |  |  |  | 0.26** | 0.07 |  |  |  |  | – 0.29* | 0.26 |
| Age | 0.15 | 0.11 |  | 0.35** | 0.11 |  | 0.06 | 0.10 |  | – 0.91 | 0.82 |  | – 1.30 | 0.57 |
| **Interaction items** |  |  |  |  |  |  |  |  |  |  |  |  |  |  |
| EPS-C × Age |  |  |  |  |  |  |  |  |  | 0.03 | 0.02 |  | 0.01 | 0.02 |
| SOC × Age |  |  |  |  |  |  |  |  |  |  |  |  | 0.02* | 0.01 |
| R^2^ |  | |  | 0.24 | |  | 0.26 | |  | 0.26 | |  | 0.32 | |

B: Regression coefficient; SE: Standard Error; EPS-C: Empathy process scale for Cognition; SOC: Sense of coherence; POMS- V: Profile of mood state Vigor.

***p* < *0.01*, **p* < *0.05*.

Supplemental Table 7. Result of moderated mediation analysis: indirect effect of empathy for other's positive affect on positive mood through sense of coherence moderated by age

|  | **Mediation analysis** | | | | | | | |  | **Moderated mediation** | | | | |
| --- | --- | --- | --- | --- | --- | --- | --- | --- | --- | --- | --- | --- | --- | --- |
| **Dependent variable** | POMS-V | |  | SOC | |  | POMS-V | |  | SOC | |  | POMS-V | |
|  | Step 1 | |  | Step 2 | |  | Step 3 | |  | Step 4 | |  | Step 5 | |
| **Independent variable** | B | SE |  | B | SE |  | B | SE |  | B | SE |  | B | SE |
| Constant | 24.11** | 6.62 |  | 9.35 | 7.12 |  | 22.05 | 6.01 |  | 26.12 | 29.92 |  | 71.82** | 18.97 |
| EPS-P | 0.44** | 0.14 |  | 0.85** | 0.16 |  | 0.25 | 0.14 |  | 0.43 | 0.73 |  | –0.14 | 0.53 |
| SOC |  |  |  |  |  |  | 0.22** | 0.08 |  |  |  |  | – 0.34 | 0.25 |
| Age | 0.12 | 0.10 |  | 0.31** | 0.12 |  | 0.05 | 0.10 |  | – 0.17 | 0.89 |  | – 1.35** | 0.49 |
| **Interaction items** |  |  |  |  |  |  |  |  |  |  |  |  |  |  |
| EPS-P × Age |  |  |  |  |  |  |  |  |  | 0.01 | 0.02 |  | 0.01 | 0.01 |
| SOC × Age |  |  |  |  |  |  |  |  |  |  |  |  | 0.02* | 0.01 |
| R^2^ |  | |  | 0.34 | |  | 0.27 | |  | 0.34 | |  | 0.34 | |

B: Regression coefficient; SE: Standard Error; EPS-P: Empathy process scale for other’s Positive affects; SOC: Sense of coherence; POMS-V: Profile of mood state Vigor.

***p* < *0.01*, **p* < *0.05*.

Supplemental Table 8. Result of moderated mediation analysis: indirect effect of empathy for other's negative affect on positive mood through sense of coherence moderated by age

|  | **Mediation analysis** | | | | | | | |  | **Moderated Mediation analysis** | | | | |
| --- | --- | --- | --- | --- | --- | --- | --- | --- | --- | --- | --- | --- | --- | --- |
| **Dependent variable** | POMS-V | |  | SOC | |  | POMS-V | |  | SOC | |  | POMS-V | |
|  | Step 1 | |  | Step 2 | |  | Step 3 | |  | Step 4 | |  | Step 5 | |
| **Independent variable** | B | SE |  | B | SE |  | B | SE |  | B | SE |  | B | SE |
| Constant | 24.62** | 6.17 |  | 26.38** | 7.84 |  | 18.21** | 6.15 |  | 100.88 | 26.49 |  | 72.27** | 20.00 |
| EPS-N | 0.48* | 0.15 |  | 0.45* | 0.19 |  | 0.37* | 0.14 |  | – 1.53* | 0.70 |  | – 0.29 | 0.52 |
| SOC |  |  |  |  |  |  | 0.25** | 0.07 |  |  |  |  | – 0.25 | 0.23 |
| Age | 0.09 | 0.11 |  | 0.29* | 0.13 |  | 0.02 | 0.09 |  | –1.89** | 0.75 |  | – 1.46* | 0.53 |
| **Interaction items** |  |  |  |  |  |  |  |  |  |  |  |  |  |  |
| EPS-N × Age |  |  |  |  |  |  |  |  |  | 0.06** | 0.02 |  | 0.02 | 0.01 |
| SOC × Age |  |  |  |  |  |  |  |  |  |  |  |  | 0.01* | 0.01 |
| R^2^ |  | |  | 0.21 | |  | 0.30 | |  | 0.26 | |  | 0.35 | |

B: Regression coefficient; SE: Standard Error; EPS-N: Empathy process scale for other’s Negative affects; SOC: Sense of coherence; POMS-V: Profile of mood state Vigor.

***p* < *0.01*, **p* < *0.05*.
